# Use of Virtual Reality in Patients with Acquired Brain Injury: A Systematic Review

**DOI:** 10.3390/jcm12247680

**Published:** 2023-12-14

**Authors:** Andrea Calderone, Diamante Carta, Davide Cardile, Angelo Quartarone, Carmela Rifici, Rocco Salvatore Calabrò, Francesco Corallo

**Affiliations:** IRCCS Centro Neurolesi Bonino-Pulejo, S.S. 113 Via Palermo, C.da Casazza, 98124 Messina, Italy

**Keywords:** acquired brain injuries, traumatic brain injuries, cognitive rehabilitation, neurorehabilitation, virtual reality

## Abstract

Background and Objectives: ABI is found in all societies as the most severe, disabling neurological disorder. A cognitive rehabilitation program is essential for the clinical recovery of these patients, improving functional outcomes and quality of life. Modern technologies such as virtual reality (VR) offer several advantages over traditional therapies, including the ability to engage people in simulated performance of functional tasks. This review will examine the studies in which virtual reality has been used as an aid, technique, or intervention in patients with acquired brain injury. Materials and Methods: Studies were identified from an online search of PubMed, Cochrane Library, and Web of Science databases. Results: We found that TBI patients responded positively to VR treatment depending on the damaged or impaired cognitive and motor functions they acquired. It is now a tool that is available in the rehabilitation of these patients and supports the recovery of various motor and cognitive functions. Conclusions: This review has shown that VR is an intervention technique that increasingly exists in clinical rehabilitation practice for ABI patients. The device uses advanced technologies that can cause general changes in cognitive, motor, and psychological aspects and create a simulated environment that can partially restore these functions and behaviors, as well as the behaviors of everyday life.

## 1. Introduction

Acquired brain injury (ABI) represents the main cause of death and impairment among young adults. It is an injury to the brain that is not hereditary, congenital, degenerative, or caused by birth trauma, as it occurs after birth. The injury alters the neuronal activity of the brain, affecting the physical integrity of the brain, the metabolic activity, or the functioning of the nerve cells in the brain. Traumatic brain injury (TBI) is the most common type of ABI (excluding stroke), with motor vehicle accidents and falls being the most common causes [1,2]. TBI is characterized as “an alteration in brain function, or other evidence of brain pathology, caused by an external force” [3] and is present in all societies as the most serious and disabling neurological disorder for more than 57 million patients worldwide [4]. Brain injuries can be divided into two main categories according to their cause: traumatic brain injury and non-traumatic brain injury. In TBI, brain damage is usually caused by direct or transmitted external influences such as falls, vehicle collisions, sports injuries, abuse/assault, and pressure explosions. In non-traumatic brain injury, brain damage is mainly caused by infection, brain tumors, ischemia, and stroke. In surgical cases, brain tissue may be inadvertently damaged by invasive procedures [5,6]. The incidence of TBI peaks in both young and late adulthood and is one of the most common causes of morbidity and mortality in young adults under 45 years of age [7]. The increased mortality risk associated with this serious condition has been shown to persist for at least seven years after the initial injury; survivors of TBI tend to develop long-term disability, negatively affecting not only the individual but also caregivers and society as a whole [8].

This clinical picture is not an isolated injury but a long-term disease process that creates a moving target for any specific treatment. Longitudinal processes after ABI include cell death, network destruction, network reorganization, immunological responses, formation of abnormal neuronal networks, glial reactions, and amyloid deposition, as well as many other mechanisms. These neurobiological mechanisms can persist for many years and occur unpredictably [9]. Individual personality traits and psychological characteristics can influence injury healing. TBI is traditionally classified using injury severity scores. The most widely used scoring system in the acute phase is the Glasgow Coma Scale (GCS), which consists of three categories (open-eye response, verbal response, and motor response) and is administered within 24 h of injury. It is performed within 24 h of injury to assess impairment of consciousness and coma. Scores range from 3 to 15, with higher scores indicating better function [10]. The clinical picture is characterized by different dysfunctions in different brain regions depending on the affected area, leading to many cognitive, motor, emotional, and neurobiological symptoms. However, cognitive function can be partially restored even in damaged brain regions thanks to neuroplastic mechanisms in the brain and can be stimulated by rehabilitation using modern technologies such as VR.

### VR Rehabilitation

The term virtual rehabilitation is used to describe a form of training in which the patient interacts with a virtual or augmented environment delivered with the help of technology [11]. Virtual rehabilitation can engage individuals in simulated practice of high-dose, functional tasks compared to traditional treatments [12], with automatic assessment of performance over time, flexibility in scaling task constraints, various reward structures to maintain compliance, and many other advantages [13]. One of the most important tools used in virtual rehabilitation is VR. It is a computer technology that refers to a computer-generated artificial environment with special sensory characteristics that can be interacted with in real time [14]. The benefits of this tool include the expectation of active learning during motivational activities, the ability to control the difficulty of the task, and the ability to measure individual behavior and performance. It can also be adapted to an individual’s specific treatment goals to provide repetitive exercises with less guidance from a clinical therapist or to gradually increase the difficulty of a task [15]. Cognitive and motor rehabilitation programs are an integral part of the clinical recovery of TBI patients and improve functional outcomes and quality of life. They are based on restorative and compensatory strategies and adapt to the patient’s resources and disability [16]. They should provide goals and opportunities to practice real and meaningful tasks to maximize function and enable engagement outside the clinical setting [17].

For example, cognitive, physiotherapy, and communication therapy goals should be implemented in everyday life [18] and relevant community settings (home, café, workplace).

The aim of this review is to examine studies using VR as a tool, technique, or intervention in patients with ABI.

## 2. Materials and Methods

### 2.1. Search Strategy

**Figure 1:**
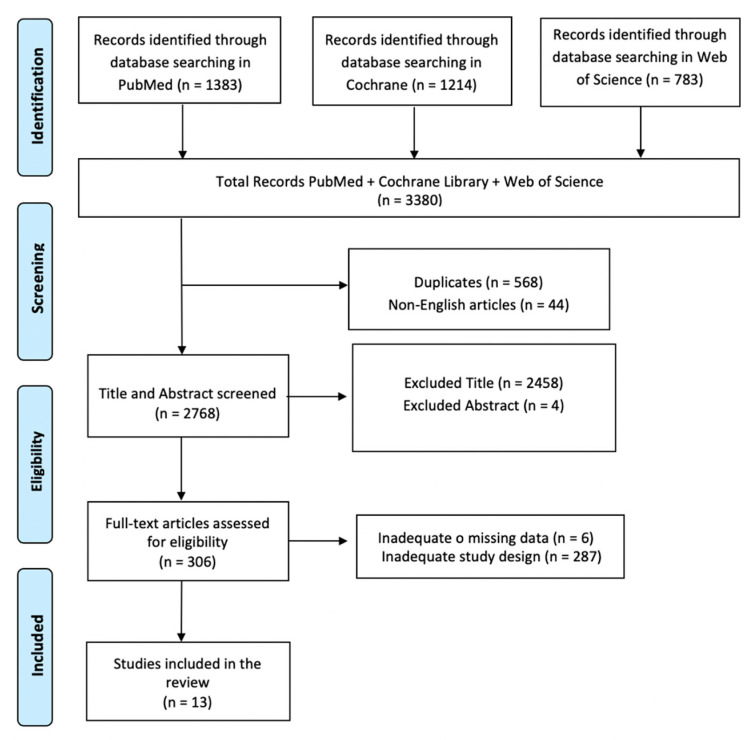
PRISMA flow chart for the current review.

A review of currently published studies was carried out for articles in which VR was used as a tool, technique, or intervention in patients with ABI. The literature search was conducted via PubMed, Cochrane Library, and Web of Science, and it was carried out for articles using the following search keyword terms: ((((ALL = (acquired brain injuries)) OR ALL = (traumatic brain injuries)) AND ALL = (cognitive rehabilitation)) OR ALL = (neurorehabilitation)) AND ALL = (virtual reality). We adopted the PRISMA (Preferred Reporting Items for Systematic Reviews and Meta-Analyses) flow diagram to describe the sequence of steps (identification, screening, eligibility, and inclusion) for the collection and identification of eligible studies, as shown in Figure 1. To avoid bias, several expert teams worked together, selected the articles, analyzed the data independently, and discussed any discrepancies with each other.

### 2.2. Inclusion Criteria

A study was included if it described or investigated the use of VR in ABI patients during cognitive or neurorehabilitation. Only articles written in English were included in the review.

### 2.3. Exclusion Criteria

A study was excluded if there was a lack of data or information about the use of VR in ABI patients. Systematic, integrative, or narrative reviews were also excluded, although their reference lists were reviewed and included if appropriate. All articles written in languages other than English were excluded.

## 3. Results

In total, 3380 articles were searched. 568 articles were removed due to duplication after screening, and 44 articles were excluded because they were not present in English. 2462 articles were removed based on title and abstract screening. Finally, 293 articles were removed based on screening for inadequate study designs and untraceable articles. Thirteen research articles met the inclusion criteria. A summary of these studies is shown in Table 1.

The articles in this review have investigated the use of VR as a tool, technique, or intervention in ABI patients. Aspects of neurological rehabilitation were also considered an integral part of the patient’s cognitive experience.

VR rehabilitation of brain injury patients was evaluated in five articles [19,20,21,22,23]. The effectiveness and verification of VR in rehabilitation programs with TBI patients were evaluated in six other articles [24,25,26,27,28,29]. VR rehabilitation in stroke patients has been discussed in an article [30]. The evaluation of IADL using VR has been evaluated in the last article [31].

### VR Rehabilitation in ABI Patients: The Use of Virtual Environments

The inclusion of VR in rehabilitation can provide a more economical method of training that reduces contact with one-on-one therapists, as well as an objective and accurate measurement of the patient’s response to a series of repetitive tasks. Since virtual reality provides a safe and changeable environment for learning, it can bring an approach to cognitive rehabilitation for those who experience ABI. The simulated environment has been proven to have good reliability and parallel validity for assessing cognitive function in patients with brain injury [19]. The VR exercise on social problem solving emphasized that as the degree and source of relevant and unrelated information increases, group differences in social problem solving become more pronounced in children with TBI [20]. In addition, sex differences have been found in the use of VR recovery of cognitive function in these patients. Women who received VR training showed better cognitive improvement. This type of training not only improved mood but also produced cognitive flexibility and selective attention. All this was influenced by sex [21]. Exercising in a virtual environment offers the potential for significant improvements in cognitive function. Combining physical activity with VR has been proven to be an effective treatment for improving cognitive function. In one study, patients with brain injuries performed significantly better in the control of numerical symbols and verbal and visual learning tasks. Significant improvements in reaction time and exercise duration were achieved after a single VR exercise [22]. A specific virtual kitchen environment was used to evaluate selective cognitive function in patients after brain injury. The computer-generated VR environment has proven to be a reproducible tool for assessing selected cognitive functions and can be used in addition to traditional rehabilitation assessments of people with ABI [23].

Another study suggests that VRRS is a suitable alternative or complementary tool, or both at the same time, to improve motor (levels of functional independence) and cognitive (frontal/executive abilities) outcomes, reduce behavioral changes (symptoms of anxiety and depression) in patients with SABI, and also has a beneficial effect on pain relief for caregivers, reducing pain and encouraging positive aspects of compassion [24]. Another program, such as Virtual Reality Shopping Tasks (VRST), demonstrates its environmental value and is a sensitive indicator of future memory dysfunction after TBI [25]. It was found that the use of this technique is useful in the neural rehabilitation of these patients with the improvement of various cognitive functions and mood, such as cognitive flexibility, attention switching, visual search, as well as executive and visual–spatial functions necessary for planning and managing daily life [26]. The inclusion of artificial intelligence software in the program of the professional training system for psychological education was also applied. They used this new system to show the viability of improving problem-solving skills and improving professional outcomes for people with TBI [27]. VR has also been used in the rehabilitation of children with brain injuries, proving its effectiveness and validity. In particular, the effect of VR training on the body function of the upper extremities using three-dimensional upper extremity kinematic assessment is a reliable and accurate tool for objectively measuring changes in joint kinematics after treatment [28]. VR programs can also be used to allow patients to experience real-life situations in which certain skills are required to perform tasks that require cognitive skills, such as memory and executive functions. In fact, future memory has been shown to improve both real-life post-rehabilitation and virtual reality, as well as various cognitive traits such as frontal lobe function and semantic fluency. VR-based training may be well accepted by ABI patients as it shows promising developments [29]. The real-world environment of virtual games also suggests the use of new therapeutic techniques that respond better and support neurological rehabilitation in patients with stroke or right brain injury [30]. The latest study has shown that the use of kitchen and coffee tasks via VR is an effective tool for the IADL assessment of patients who have sustained TBI [31].

## 4. Discussion

Our review aims to analyze studies in which VR has been used as a tool, technique, or intervention in patients with ABI. All the studies included in this review have shown that VR is more effective in ABI patients who promote improvements in cognition (positive memory, executive function, attention), exercise, and society (activities of daily living). It is widely used and functional in the rehabilitation of cognitive function in patients with ABI. Improvements were found in the following areas: attention, flexibility, symbol learning, and execution abilities [19,20,21,22,23]. The tool also improved the emotional space of these patients in terms of mood and reduced the symptoms of anxiety and depression [24]. These studies found sex differences in which cognitive improvement was better in women than in men [21]. Finally, these VR programs have the property of being useful in assessing cognitive impairment and different simulated environments. They reproduce the daily environment and promote greater commitment and recovery during rehabilitation [25,26,27,28,29,30,31]. We found virtual kitchens, cafes, virtual shopping malls, and virtual games among the main life simulation environments used in virtual rehabilitation [21,22,23,31]. Each of them has certain characteristics and objects that remind of cognitive, motor, and social functions that can be rehabilitated in certain continuous sessions. We know that VR technology offers the opportunity to develop human performance testing and educational environments [32]. These virtual surroundings can present the illusion of three-dimensional space. Participants with limited motor control can safely “move” and engage in a range of activities in a simulated environment, relatively far from the restrictions imposed by obstacles. It provides a high degree of control and, at the same time, integrates various sensory functions simultaneously. Since the computer system calculates and records changes over time, the evaluation in the virtual world can be carried out objectively with minimal human error and bias [33]. Exercises performed in a virtual environment allow the patient to understand the consequences of movements (knowledge of results) and the quality of movements (knowledge of performance), which positively affect the functional recovery of the patient, including cognitive ones. Some studies based on VR for cognitive assessment of individuals with brain injury have shown that the technology is able to detect disturbances in daily living activity and that the simulated environment is a good predictor of daily living function [34,35].

However, we also need to take into account that TBI damage can interfere with physical ability, cognition, and emotional state, depending on the injured area [36]. These disorders may leave a residual deficiency that may prevent the injured person from participating in activities of daily living (ADL). This involves multiple cognitive and motor processes (e.g., to achieve functional goals requires organization, ordering steps, object identification, and vehicle use), and healthy people can easily perform these tasks, even if they experience a hitch when moving under a large number of conditions, such as temporal pressure, or divided attention [37]. This premise was made to be able to adapt the virtual world to the abilities and needs of each individual, unlike the real world. It offers great flexibility in virtual work and experience, which includes the necessary sequence of functional movements to achieve autonomy in real-life activities. The analysis and control of the virtual component of all activities by occupational therapists allows for virtual achievements that cannot be achieved in real life due to disability or environmental restrictions. These results increase motivation and commitment to the rehabilitation process [38].

### Neurorehabilitation with VR

Cognitive and physical rehabilitation programs optimize activity, function, performance, productivity, participation, and quality of life, improving clinical outcomes in ABI patients. Rehabilitation programs are based on recovery, compensation, and adaptation strategies that vary depending on the patient’s potential and degree of disability [39]. VR provides a higher level of engagement and cognitive participation than memory and imagination provide, but also directly through “real” experiences. Its multisensory stimulation can be applied to functional and ecological real-world demands (for example, finding objects, deconstructing objects, etc.) and has the potential to improve brain plasticity and regeneration processes [40,41]. Based on the identified studies, VR technologies used for the rehabilitation of ABI patients include the immersive type (high-accuracy resolution of panoramic view and head-mounted screens, data gloves, or bodysuits are used); the non-immersive type is desktop-based (computer and large monitor, smartphone, etc.). We can assess that there are three main types of video capture VR (virtual environment created and displayed by the speaker) that are not immersive. It is different from desktop-based, video capture VR in the necessary equipment and control mode. Instead of using a desktop computer, a color key background, a large video screen, and a video camera are used in the video capture VR. These three types can be used to create a sophisticated simulated environment that can replicate the situations and events of everyday life, making the neural rehabilitation process functional and realistic. We know that TBI causes deficits in attention, memory, information processing speed, and executive function. A high level of cognitive function depends on well-functioning distributed brain networks and finely regulated neurotransmitter systems that can be destroyed by damage [42,43]. In this clinical context, VR can help to partially restore this brain network through virtual simulations, such as daily life activities. They are multitasking activities and require not only motor participation, but also cognitive participation. From this point of view, it seems even more likely that VR will play a new fundamental role in neural rehabilitation. Of all the possible applications, one of the most common is the VMall. It consists of exercises performed in a virtual environment that recreates the supermarket [44]. Simulated exercise can improve the patient’s active participation in rehabilitation performed using robots, which carries the risk of becoming practically passive [45]. At the neurobiological level, visual feedback can activate a network of cortical regions, including the upper and lower parietal cortex, ventral premotor cortex, primary motor cortex, dorsal premotor cortex, lower frontal gyrus, and auxiliary motor regions [46]. Despite these advantages, the use of VR technology in the rehabilitation of ABI patients remains limited. In a study conducted on a small number of registered patients with mild traumatic brain injury, it was found that its use led to moderate improvements in terms of gait and balance [47,48]. VR can contribute to increasing awareness of patient performance and promoting mental concentration and participation, which are all necessary elements for motor learning [49]. In this sense, the type and intensity of exercise and the context of the environment are important factors [50,51]. VR allows for a real learning process that can reconstruct the brains of ABI patients through a simulated environment and the necessary tasks, causing changes in neuronal formation. When a brain region loses some of its connections, it undergoes a cascade of changes related to the clearance of degenerating debris, the remodeling of neuronal processes, and the production of new neural connections (synapses) by remaining inputs, a process termed reactive synaptogenesis [52]. Further research could analyze and deepen the role of virtual reality in both cognitive and emotional rehabilitation by analyzing further correlations between the cognitive and perceptual learning experience and their impact on the mood or emotionality of the patient with ABI within a rehabilitation process.

## 5. Conclusions

The review shows that VR is an intervention technique that increasingly exists in the clinical rehabilitation practices of ABI patients. The device can partially restore functions and behaviors, as well as daily life behaviors, using advanced technologies that can create a simulated environment that can often cause changes in cognitive, motor, and psychological aspects. We can say that rehabilitation has become possible not only in real life but also through VR. We have observed a limited number of studies on the use of this tool in ABI patients during cognitive or neurological dysfunction (only 13 out of a total of more than 300 articles). This data could be possibly indicative of a lack of research related to the specific use of these instruments during rehabilitation, despite the number of patients with ABI constantly increasing. Other updates to this device could create new simulated environments and help open up new rehabilitation perspectives for patients diagnosed with ABI. Furthermore, subsequent studies on the effects of virtual reality rehabilitation and the plastic changes of the brain resulting from learning mechanisms could provide further developments on the cognitive reserve of patients after TBI.

## Figures and Tables

**Table 1:** Summary of studies included in the research.

Author	Aim	Study Design/Intervention	Treatment Period	Sample Size	Outcomes Measures	Main Findings
Zang et al., 2022 [19]	To establish the stability and validity of information collected from persons with TBI in a virtual reality environment.	Prospective correlation design to examine 3-week test-retest results for equivalence reliability between computer-simulated and natural environments.	3 weeks.	54 patients with TBI who received rehabilitation services and who were at different stages of recovery.	Time and error in task completion using a virtual reality assessment, actual kitchen performance (analogous to the virtual reality environment), an OT assessment, and neuropsychological testing	The VR system displays adequate reliability and validity as an assessment method for individuals with brain injury.
Hanten et al., 2011 [20]	To determine the effects of TBI on virtual reality social problem-solving tasks and relations to cortical thickness in adolescence.	A comparative study.	Not specified.	28 youths ages 12–19 years (15 with moderate to severe TBI, 13 uninjured).	Naturalistic, computerized VR version of the Interpersonal Negotiations Strategy interview.	Adolescents with TBI were significantly impaired in the summarized VR-SPS score. Significant group differences were strongest and most consistent in problem definition and outcome scores.
Bruschetta et al., 2022 [21]	To assess whether demographic and clinical variables might be associated with recovery of cognitive function in TBI patients after well-validated VR training.	A secondary analysis of data from a prospective randomized controlled trial.	From January 2016 to December 2018.	100 patients with TBI.	All patients were assessed before and after the end of training with a complete neuropsychological battery.	VR-induced improvement in mood, cognitive flexibility, and selective attention was influenced by sex. Women who had participated in VR training showed better cognitive recovery.
Grealy et al., 1999 [22]	Evaluation of the impact of exercise and VR on cognitive rehabilitation of individuals with TBI.	Comparative study.	4-week program.	A consecutive sample of 13 suitable TBI adults.	Tests on attention, information processing, learning, and memory. Reaction and movement times.	Training in a virtual environment offers the potential for a significant improvement in cognitive functions.
Zhang et al., 2001 [23]	Assessment of selected cognitive functions of individuals with TBI using a computer-simulated virtual reality environment.	A clinical trial.	Ranged from 14 to 5061 days.	30 patients with brain injury and 30 volunteers without brain injury.	The assessment was based on the number of correct answers and the time taken to complete everyday tasks.	A computer-generated VR environment provides a reproducible tool for assessing selected cognitive functions and can be used as an adjunct to traditional rehabilitation assessment in individuals with ABI.
Calabrò et al., 2023 [24]	Testing the efficacy of advanced training with a non-immersive VRRS to improve functional outcomes in patients with SABI.	Multicenter randomized controlled trial.	From October 2018 to August 2022.	40 patients with SABI and their 40 caregivers visiting 2 Italian rehabilitation centers were enrolled in the study protocol and randomized into 2 groups.	BI, TS, MAS, MoCa, FAB, BDI-II, SF-36, and PGWBI.	The VRRS is a suitable alternative or complementary tool, or both, to improve motor and cognitive outcomes and reduce behavioral changes in patients with SABI, which also positively impacts managing caregiver burden by alleviating stress and promoting positive aspects of care.
Canty et al., 2014 [25]	Evaluation of the sensitivity, convergent validity, and ecological validity of a newly developed PM task in virtual reality for use with individuals with TBI.	A comparative study.	The total duration of administration was about two hours, with some participants being tested in two sessions if they were prone to fatigue.	30 individuals with TBI and 24 uninjured adults matched on age, sex, and education level were administered.	The VRST, a lexical decision PM task, an index of task-friendliness, and a cognitive assessment battery.	Performance on the VRST significantly predicted caregiver ratings of patients’ occupational activities and independent living skills.
De Luca et al., 2019 [26]	Evaluation of the effects of VR training BTs-N on the recovery of cognitive functions in subjects with TBI using the interactive semi-immersive program.	A randomized controlled trial.	8 weeks.	100 patients with TBI.	MoCA, HRS-D, HRS-A, FAB, WT, VS, TMT.	VRTG and TCRG showed significant improvement in cognitive function and mood, but only VRTG showed significant improvement in cognitive flexibility and shifting abilities as well as selective attention.
Man et al., 2013 [27]	Investigating the effectiveness of an artificial intelligence-based VR vocational problem-solving skills training program to improve employment opportunities for people with TBI.	A randomized controlled trial.	12 sessions of 20–25 min, with 3 follow-ups (1 month, 3 months, 6 months).	40 patients with brain injury.	WCST, TOL, and VCRS.	An improvement in selective memory processes and the perception of memory function was observed. A cross-group comparison showed that the VR group performed better on objective and subjective outcome measures than the therapist-led group and achieved better occupational outcomes.
Choi et al., 2021 [28]	Investigating the efficacy of a VR rehabilitation system with wearable multi-inertial sensors to improve upper limb function in children with brain injury.	Randomized controlled trial.	4 weeks.	80 children (39 boys, 41 girls) with brain injury including cerebral palsy aged 3 to 16 years.	Both functional and kinematic assessments were performed for all patients at baseline (within 72 h before intervention), at the end of the fourth week intervention.	Both virtual reality rehabilitation and conventional occupational therapy were effective in upper limb training. VRT was superior in improving dexterity, performance of activities of daily living, and active supination movement of the forearm. The effect of VRT was significant in children with more severe motor impairments.
Yip et al., 2013 [29]	Determining the effectiveness of the VRPM training program for everyday PM compared to controls.	Randomized controlled trial.	12 sessions VRPM training program. It was conducted twice a week and each session lasted about 30 to 45 min (depending on the difficulty of the tasks and the abilities of the individual participants).	37 subjects.	MMSE-CV was used to screen out those with severe cognitive impairment. TONI-3 was developed to be a non-verbal abstract problem-solving test. and SADI-CV.	Both VR-based and real PM results showed significantly better changes in cognitive attributes such as frontal lobe functions and semantic fluency. VR-based training can be well accepted by ABI patients.
Fernandes et al., 2014 [30]	Comparison of the immediate effect of training with a virtual reality game in stroke patients depending on the side of the brain injury.	Comparative study.	Two series of 10 tries of 45 s, with 15 min rest between them, a total of 30 min.	The participants included 20 patients (10 right brain injury), mean age of 50.6 ± 9.2 years, and 20 healthy subjects of 50.9 ± 8.8 years.	All participants performed a kinematic evaluation of drinking a cup of water before and after training with the XBOX 360 Kinect^®^ (Microsoft, Redmond, WA, USA) table tennis game, in two series of 10 trials of 45 s, with 15 min rest in between, for a total of 30 min.	Patients with a right hemisphere injury responded better to the VR game, indicating the introduction of new treatment techniques to promote neurorehabilitation.
Besnard et al., 2016 [31]	To examine the use of the NI-VCT for IADL assessment in patients with TBI.	A quasi-experimental research design.	Two training sessions on using the virtual environment before the test session. The training sessions lasted until the participants were familiar with the devices (maximum 10 min).	19 Patients with TBI and 24 healthy controls.	TMT, MCST, TOL, and ST.	The virtual kitchen is a valid tool for the assessment of IADL in patients who have suffered from TBI.

Legend: Occupational therapy (OT), Virtual Reality-Social Problem Solving (VR-SPS), Virtual Reality Rehabilitation System (VRRS), Severe Acquired Brain Injury (SABI), Barthel Index (BI), Tinetti Scale (TS), Modified Ashworth Scale (MAS), Montreal Cognitive Assessment (MoCa), Frontal Assessment Battery (FAB), Beck Depression Inventory II (BDI-II), Short Form Health Survey 36 (SF-36), Psychological General Well-Being Index (PGWBI), Caregiver Burden Inventory (CBI),, Perspective Memory (PM), Virtual Reality Shopping Task (VRST), BTs-Nirvana (BTs-N), Hamilton Rating Scale Depression (HRS-D), Hamilton Rating Scale Anxiety (HRS-A), Weigl’s Test (WT), Visual Search (VS), Trail Making Test (TMT), Virtual Reality Training Group (VRTG), Traditional Cognitive Rehabilitation Group (TCRG), Wisconsin Card Sorting Test (WCST), Tower of London (TOL), Vocational Cognitive Rating Scale (VCRS), Virtual Reality Training (VRT), Virtual Reality-Based Prospective Memory (VRPM), Prospective Memory (PM), Chinese Version Mini Mental State Examination (MMSE-CV), Test of Nonverbal Intelligence—3rd Edition (TONI-3), Chinese Version of the Self-Awareness of Deficit Interview (SADI-CV), Non-immersive Virtual Coffee Task (NI-VCT), Instrumental Activities of Daily Living (IADL), Modified Wisconsin Card Sorting Test (MCST), Stroop Test (ST).

## Data Availability

The data that support the findings of this study are not openly available due to reasons of sensitivity and are available from the corresponding author upon reasonable request.
